# Cognitive Profile Subgroups Among Non‐demented Older Adults and their Associations with AD Neuroimaging Measures

**DOI:** 10.1002/alz.091667

**Published:** 2025-01-03

**Authors:** Alice M Hahn, Heather E Volk, Marilyn S. Albert, Anja Soldan, The BIOCARD Research Team

**Affiliations:** ^1^ Johns Hopkins Bloomberg School of Public Health, Baltimore, MD USA; ^2^ Johns Hopkins University School of Medicine, Baltimore, MD USA

## Abstract

**Background:**

Although it is well established that lower cognitive performance, on average, is associated with a greater risk of developing Alzheimer’s disease (AD) dementia, it is unclear whether distinct cognitively‐defined subgroups exist among non‐demented older adults and whether such profiles map onto distinct AD neuroimaging measure profiles.

**Method:**

The sample consisted of 167 non‐demented older adults from the BIOCARD study with comprehensive neuropsychological and clinical evaluations, amyloid PET and brain MRI scans. The MRI measure included: global cortical volume in AD‐signature regions and a medial‐temporal lobe composite; resting‐state functional connectivity within 5 large‐scale cognitive networks; global white matter microstructure, index by fractional anisotropy (FA) and radial diffusion (RD) on DTI scans; and global white matter hyperintensity (WMH) volume on FLAIR scans. A latent profile analysis (LPA) identified cognitively defined subgroups using standardized scores of 8 cognitive tests that cover AD‐related domains (episodic memory, executive functions, working memory, semantic memory, language, visuospatial function, and attention). Multiple linear regression models tested the associations between the group membership and neuroimaging measures adjusting for age, sex, and education.

**Result:**

The LPA identified a model with 3 subgroups as the best solution (AIC = 3515; BIC = ‐3605; ICL = ‐3642; BLRT *p* = 0.001). The groups primarily differed in terms of performance level: high (36%); average (53%); low (11%). Each group showed distinctive diagnosis composition: high ─ 100% cognitively normal (CN); average ─ 91% CN and 9% mild cognitive impairment (MCI); low ─ 61% CN and 39% MCI. Compared to the high performance group, the low performance group had significantly lower cortical volume in AD vulnerable regions, lower salience/ventral attention network functional connectivity, lower global white matter microstructural integrity, measured by FA and RD, and higher global WMH burden. Amyloid load was not associated with group membership.

**Conclusion:**

Distinct subgroups defined by cognitive performance exist among non‐demented older adults that are related to AD neuroimaging measures. Evaluating cognitive profiles among non‐demented older individuals may help identify individuals at greatest risk for future cognitive decline.

